# Ziemssen’s Cyclopædia of the Practice of Medicine

**Published:** 1875-02

**Authors:** 


### Books Reviewed.

*Cyclopædia of the Practice of Medicine.* Edited by Dr. H. von Ziemssen, Vol. I, Acute Infectious Diseases. By Profs. Liebermeister, Lebert and Heubner, Drs. Haenisch and Oertel. The American Translation edited by Albert H. Buck, of New York. New York: Wm. Wood & Co., 1874.

The first volume of Ziemssen's Cyclopedia which has been anticipated for some time by the profession, has been on our table for a considerable period, and we are even now unable to give it that thorough consideration which its importance demands.

The first article is by Prof. Liebermeister upon Infectious Diseases, followed by a long article by the same author upon Typhoid Fever. The writer evidently speaks from a large experience both in the literature of the disease and with the disease itself. The treatment which he adopts would seem rather heroic to us, but may be perhaps the thing when the patient is a phlegmatic German. An article upon the Plague is by the same writer, and is chiefly interesting in a historical point of view. Relapsing Fever, Typhus Fever and Cholera, by Prof. Lebert, Yellow Fever by Dr. Haenisch, Dysentery, by Prof. Heubner, and Epidemic Diphtheria, by Dr. Oertel, compose the balance of the work. We regret that our space will not allow a more extended notice of these articles, that upon Diphtheria is of especial interest at this time, and is a valuable resumé of the subject. The work of translating is well done, and the separate indexing of each volume is an admirable idea. The Publishers deserve much praise for the enterprise, and the promptness with which they have issued the second volume.

